# Hematological Indices and Abnormalities in Chronic Kidney Disease and Their Associations With Disease Severity

**DOI:** 10.7759/cureus.94222

**Published:** 2025-10-09

**Authors:** Ukasha Tahir, Hira Akram, Rida Mahmood, Zunaira Nisar, Hafiz Zunair Iqbal, Ateeqa Sundas Gulfeshan, Mubeshar Hassan, Noor Ul Ain, Tayyaba Arooj Mufti, Adeel Ahmed, Muhammad Irfan Jamil

**Affiliations:** 1 Internal Medicine, Ittefaq Hospital (Trust) Lahore, Lahore, PAK; 2 Emergency Medicine, Nisar Medical Complex Hospital, Rahim Yar Khan, PAK; 3 Nephrology, Pakistan Kidney and Liver Institute and Research Centre, Lahore, PAK; 4 Internal Medicine, Akhtar Saeed Medical and Dental College, Lahore, PAK; 5 Anaesthesia, University Hospital Waterford, Waterford, IRL; 6 Medicine, Combined Military Hospital (CMH) Lahore Medical College and Institute of Dentistry, Lahore, PAK; 7 Trauma and Orthopaedics, Forth Valley Royal Hospital, Larbert, GBR; 8 Urology, Shifa Hospital, Sargodha, PAK; 9 Internal Medicine, Rawal Institute of Health Sciences, Islamabad, PAK; 10 Cardiology/Medicine, Akhtar Saeed Medical and Dental College, Lahore, PAK; 11 Anaesthesiology and Critical Care, King Edward Medical University, Lahore, PAK; 12 Nephrology, Lahore General Hospital, Lahore, PAK

**Keywords:** anemia in ckd, chronic kidney disease (ckd), hematological abnormalities, leukocytosis, thrombocytopenia

## Abstract

Background and objective: Chronic kidney disease (CKD) is frequently complicated by hematological abnormalities, including anemia, thrombocytopenia, and leukocyte disorders, which significantly impact morbidity and survival. This study aimed to evaluate hematological indices and abnormalities in patients with CKD and determine their associations with disease severity across stages 3-5.

Methods: This cross-sectional observational study was carried out in the Department of Nephrology, Lahore General Hospital, Pakistan, between August 2023 and February 2024, following Institutional Review Board approval. A total of 412 patients aged 18-70 years with CKD stages 3-5, defined by Kidney Disease: Improving Global Outcomes (KDIGO) criteria, were recruited using non-probability consecutive sampling after obtaining written informed consent. Patients with known hematological disorders, acute infections, malignancy, chronic hepatitis B or C, HIV, recent blood transfusion, use of erythropoiesis-stimulating agents, or pregnancy were excluded. Venous blood samples (10 mL) were collected under aseptic precautions and analyzed for hematological indices using the Sysmex KX-2 analyzer (Kobe, Japan). Biochemical parameters were measured on Cobas Integra 400 Plus (Roche Diagnostics, Germany).

Results: A total of 412 patients were evaluated, with a mean age of 44.26 ± 14.05 years; 181 (43.9%) were aged 18-45 years, and 231 (56.1%) were 46-70 years. Men comprised 266 (64.6%) and women 146 (35.4%). The mean disease duration was 4.93 ± 2.61 years, with 247 (60%) reporting <5 years. Diabetes mellitus (40%) and hypertension (31.3%) were the leading etiologies. CKD stages included 100 (24.3%) in stage 3, 169 (41%) in stage 4, and 143 (34.7%) in stage 5. Hemoglobin (Hb) declined from 12.65 ± 1.05 g/dL (stage 3) to 9.98 ± 1.97 g/dL (stage 5, p < 0.001). Hematocrit (Hct) decreased from 35.61 ± 4.33% to 29.20 ± 5.45%, red blood cell (RBC) count from 4.04 ± 0.74 × 10¹²/L to 3.38 ± 0.50 × 10¹²/L, and platelet (PLT) count from 272.15 ± 116.68 × 10⁹/L to 197.82 ± 130.32 × 10⁹/L. Red cell distribution width (RDW) increased from 13.58 ± 1.37% to 15.79 ± 1.50% (p < 0.001). Anemia was present in 38%, 68%, and 92.3% of stages 3, 4, and 5, respectively; thrombocytopenia (14%-34.3%), leukopenia (11%-25.2%), and leukocytosis (7%-22.4%) also increased significantly with disease severity.

Conclusion: Hematological indices deteriorate with advancing CKD, with anemia, thrombocytopenia, and leukocyte abnormalities showing strong associations with disease severity. Routine hematological monitoring is essential for early detection and management of these complications to mitigate disease-related morbidity and improve patient outcomes.

## Introduction

Chronic kidney disease (CKD) is a major public health problem worldwide, with prevalence estimates ranging between 8% and 16% in the adult population [1]. The global prevalence is projected at 13.4%, and the number of patients requiring renal replacement therapy for end-stage renal disease is expected to reach between 4.9 and 7.1 million [2,3]. Epidemiological studies from South Asia show CKD prevalence of 13%-15% in India and up to 26% among adults in Bangladesh, while surveys in rural communities have identified nearly one-third of individuals with undiagnosed disease [4]. In Pakistan, the prevalence of CKD has been reported at 21.2%, underscoring the significant national burden [5]. The rising trend is driven by the increasing prevalence of non-communicable diseases such as obesity, hypertension, and diabetes. CKD is defined by the abnormalities of kidney structure or function lasting at least three months, with progressive decrease in glomerular filtration rate (GFR) often occurring silently until advanced stages when dialysis or transplantation becomes necessary [6].

CKD is accompanied by multiple systemic complications, including electrolyte disturbances, metabolic acidosis, anemia, hypertension, malnutrition, bone mineral disorders, and hormonal abnormalities [7]. Hematological changes are among the most consistent, with anemia being the predominant abnormality. The pathogenesis of anemia is multifactorial, but reduced erythropoietin production remains central, compounded by shortened red cell survival, hemolysis, iron deficiency, vitamin B12 and folate deficiency, chronic inflammation, gastrointestinal or dialysis-related blood loss, and resistance to erythropoietin [8,9]. Red cell indices such as hemoglobin (Hb), hematocrit (Hct), mean corpuscular volume (MCV), mean corpuscular hemoglobin (MCH), mean corpuscular hemoglobin concentration (MCHC), and red cell distribution width (RDW) show progressive alterations with declining kidney function [10]. Swarnalatha and Vinotha demonstrated significant reductions in red blood cell (RBC) counts, total leukocyte counts, and platelets (PLT) across CKD stages 3, 4, and 5, with normocytic normochromic anemia being the most frequent blood picture [11]. Another study reported anemia in 86.7% of patients, along with leucopenia in 15.2%, leukocytosis in 6.7%, thrombopenia in 23.8%, and thrombocytosis in 3.8%, highlighting the broad hematological spectrum in CKD [12].

Beyond red cell abnormalities, changes in white blood cells (WBCs) and platelets are also recognized. Leukocyte dysfunction contributes to immune suppression and increased susceptibility to infection, while the neutrophil-to-lymphocyte ratio (NLR) has emerged as a marker of systemic inflammation and cardiovascular risk in CKD. Platelet dysfunction, characterized by impaired aggregation and adhesiveness, contributes to bleeding diathesis [13]. Collectively, these hematological disturbances contribute to fatigue, reduced quality of life, increased cardiovascular morbidity, higher infection risk, and poorer survival [14]. The frequency and severity of these abnormalities vary across CKD stages and may differ according to underlying causes such as diabetic kidney disease, hypertensive nephrosclerosis, glomerulonephritis, obstructive uropathy, and polycystic kidney disease [15].

Given this background, evaluating hematological indices and abnormalities in patients with CKD and assessing their associations with disease severity is essential. Such an approach will not only quantify the burden of hematological complications but also provide insight into mechanisms leading to morbidity in different patient subgroups. The objective of the present study is therefore to examine hematological indices and abnormalities in patients with CKD and to identify their associations with disease severity.

## Materials and methods

This cross-sectional observational study was conducted in the Department of Nephrology at Lahore General Hospital, Lahore, Pakistan, from August 2023 and February 2024, following authorization from the Institutional Review Board (approval number: 161/08/2023). A non-probability consecutive sampling was used to enroll patients. A sample size of 412 was calculated using an estimated prevalence of leukocytosis in CKD of 19.2%, with an absolute precision of 5% and a confidence level of 99% [16].

Patients of either sex aged 18-70 years with CKD stages 3-5, defined according to Kidney Disease: Improving Global Outcomes (KDIGO) criteria [17], who provided informed consent were included. Exclusion criteria comprised known hematological disorders, acute infections, malignancy, chronic hepatitis B or C, HIV, tuberculosis, recent blood transfusion or major bleeding within three months, use of erythropoiesis-stimulating agents or iron therapy within four weeks, treatment with bone marrow-suppressive drugs, and pregnancy.

Prior to participation, all patients were informed in detail about the objectives, procedures, risks, and possible benefits of the study in their local language, and written informed consent was obtained. Each participant was assigned a coded identification number to maintain confidentiality, and no personal identifiers were stored in the study database. Baseline demographic and clinical information was obtained at enrollment through a structured interview and review of medical records. This included age, gender, and duration of disease. The underlying etiology of CKD was documented and classified as diabetes mellitus, hypertension, glomerular disease, renal stone disease, or polycystic kidney disease.

For laboratory assessment, 10 mL of venous blood was drawn from each participant under aseptic conditions by trained personnel and immediately transported to the hospital laboratory. Full blood counts were performed using an automated hematology analyzer (Sysmex KX-2, Kobe, Japan). Hematological indices measured included Hb, Hct, RBC, MCV, MCH, RDW, WBC, neutrophil count, lymphocyte count, and platelet count. Biochemical parameters, including serum creatinine and blood urea, were analyzed using the Cobas Integra 400 Plus auto-analyzer (Roche Diagnostics, Germany). Estimated glomerular filtration rate (eGFR) was calculated using the CKD-EPI equation, and CKD stage was assigned accordingly as stage 3, stage 4, or stage 5. Anemia was defined as Hb < 13 g/dL in men and Hb < 12 g/dL in women. Leukopenia was defined as WBC < 4 × 10⁹/L, and leukocytosis as WBC > 11 × 10⁹/L. Thrombocytopenia was defined as PLT < 150 × 10⁹/L, while thrombocytosis was defined as PLT > 450 × 10⁹/L. Outcomes were labeled at the time of enrollment based on these laboratory thresholds, ensuring objective and reproducible classification.

Data were entered and analyzed using the Statistical Package for the Social Sciences (SPSS) version 26.0 (IBM Corp., Armonk, NY). Continuous variables were expressed as mean ± standard deviation (SD). Categorical variables were summarized as frequencies and percentages. Comparisons of mean values of hematological indices across CKD stages (3, 4, and 5) were performed using one-way analysis of variance (ANOVA). Associations between categorical hematological abnormalities and CKD stage were evaluated using Pearson’s Chi-square (χ²) test. A p-value of <0.05 was considered statistically significant.

## Results

The study population had a mean age of 44.26 ± 14.05 years, with 56.1% aged 46-70 years. Men constituted 64.6% of the participants. The mean duration of disease was 4.93 ± 2.61 years, with 60% reporting a duration of less than five years. Diabetes mellitus was the most frequent etiology (40%), followed by hypertension (31.3%), renal stone disease (15.8%), and glomerular disease (12.9%). According to KDIGO classification, 24.3% of the patients were in stage 3, 41% in stage 4, and 34.7% in stage 5 (Table 1).

**Table 1:** Baseline Demographic and Clinical Characteristics of Patients With Chronic Kidney Disease (N = 412) Values are presented as mean ± SD for continuous variables and frequency (number) with percentage (%) for categorical variables. CKD: chronic kidney disease, SD: standard deviation

Variable	Category	Frequency (number)	Percentage (%)
Age (years)	Mean ± SD	44.26 ± 14.05	-
Age group	18-45 years	181	43.9
	46-70 years	231	56.1
Gender	Male	266	64.6
	Female	146	35.4
Duration of disease	Mean ± SD	4.93 ± 2.61	-
	<5 years	247	60.0
	≥5 years	165	40.0
Etiology	Diabetes mellitus	165	40.0
	Hypertension	129	31.3
	Glomerular disease	53	12.9
	Renal stone disease	65	15.8
CKD stage	Stage 3	100	24.3
	Stage 4	169	41.0
	Stage 5	143	34.7

The mean hemoglobin level was 10.92 ± 2.05 g/dL, showing a significant decline from 12.65 ± 1.05 g/dL in stage 3 to 9.98 ± 1.97 g/dL in stage 5 (p < 0.001). Hematocrit decreased from 35.61 ± 4.33% in stage 3 to 29.20 ± 5.45% in stage 5, with an overall mean of 32.33 ± 5.56% (p < 0.001). Red blood cell counts fell progressively from 4.04 ± 0.74 × 10¹²/L in stage 3 to 3.38 ± 0.50 × 10¹²/L in stage 5 (p < 0.001). Platelet counts also declined with severity, from 272.15 ± 116.68 × 10⁹/L in stage 3 to 197.82 ± 130.32 × 10⁹/L in stage 5 (p < 0.001). MCV and MCH both decreased significantly across stages (p = 0.001 and p < 0.001, respectively), while RDW rose from 13.58 ± 1.37% in stage 3 to 15.79 ± 1.50% in stage 5 (p < 0.001). WBC counts increased significantly with advancing stage (7.18 ± 2.54 × 10⁹/L in stage 3 versus 8.23 ± 2.64 × 10⁹/L in stage 5, p = 0.005), driven by a rise in neutrophil count and a concomitant fall in lymphocyte count (p < 0.001 for both) (Table 2).

**Table 2:** Hematological Indices Across CKD Stages (N = 412) Values are presented as mean ± SD. Significance was tested using one-way ANOVA; F = test statistic. CKD: chronic kidney disease, RBC: red blood cells, PLT: platelet count, MCV: mean corpuscular volume, MCH: mean corpuscular hemoglobin, RDW: red cell distribution width, WBC: white blood cells, SD: standard deviation, ANOVA: analysis of variance

Variable	Total (mean ± SD)	Stage 3 (n = 100)	Stage 4 (n = 169)	Stage 5 (n = 143)	Test statistic (F)	p-value
Hemoglobin (g/dL)	10.92 ± 2.05	12.65 ± 1.05	10.69 ± 1.95	9.98 ± 1.97	68.15	<0.001
Hematocrit (%)	32.33 ± 5.56	35.61 ± 4.33	33.02 ± 4.93	29.20 ± 5.45	51.53	<0.001
RBC (×10¹²/L)	3.64 ± 0.67	4.04 ± 0.74	3.63 ± 0.64	3.38 ± 0.50	32.76	<0.001
Platelets (×10⁹/L)	223.87 ± 128.47	272.15 ± 116.68	217.35 ± 126.46	197.82 ± 130.32	10.70	<0.001
MCV (fL)	82.01 ± 10.46	85.14 ± 9.57	81.75 ± 9.72	80.13 ± 11.42	7.05	0.001
MCH (pg)	28.50 ± 3.51	30.17 ± 3.27	28.43 ± 2.78	27.41 ± 3.99	19.88	<0.001
RDW (%)	14.76 ± 1.79	13.58 ± 1.37	14.59 ± 1.75	15.79 ± 1.50	59.43	<0.001
WBC (×10⁹/L)	7.78 ± 2.50	7.18 ± 2.54	7.76 ± 2.28	8.23 ± 2.64	5.28	0.005
Neutrophil count	5.04 ± 0.99	4.66 ± 0.62	4.94 ± 0.90	5.42 ± 1.16	20.86	<0.001
Lymphocyte count	2.81 ± 0.55	3.03 ± 0.27	2.95 ± 0.52	2.49 ± 0.60	44.86	<0.001

Anemia was the most common abnormality, affecting 38% of the patients in stage 3, 68% in stage 4, and 92.3% in stage 5, with a strong association with advancing stage (p < 0.001). Thrombocytopenia also increased significantly, from 14% in stage 3 to 34.3% in stage 5 (p = 0.001). Thrombocytosis remained infrequent, with rates of 7%, 6.5%, and 9.1% across stages, showing no significant stage-wise association (p = 0.672). Leukopenia rose progressively from 11% in stage 3 to 25.2% in stage 5 (p = 0.019), while leukocytosis increased from 7% in stage 3 to 22.4% in stage 5, demonstrating a significant relationship with CKD severity (p = 0.001) (Table 3).

**Table 3:** Frequency of Hematological Abnormalities Across CKD Stages (N = 412) Values are expressed as frequency (number) and percentage (%). Statistical significance tested using Pearson’s Chi-square test; χ² = test statistic. CKD: chronic kidney disease

Variable	Category	Stage 3 (n = 100)	Stage 4 (n = 169)	Stage 5 (n = 143)	Chi-square (χ²)	p-value
Anemia	Present	38 (38.0%)	115 (68.0%)	132 (92.3%)	33.09	<0.001
	Absent	62 (62.0%)	54 (32.0%)	11 (7.7%)		
Thrombocytopenia	Present	14 (14.0%)	39 (23.1%)	49 (34.3%)	13.41	0.001
	Absent	86 (86.0%)	130 (76.9%)	94 (65.7%)		
Thrombocytosis	Present	7 (7.0%)	11 (6.5%)	13 (9.1%)	0.79	0.672
	Absent	93 (93.0%)	158 (93.5%)	130 (90.9%)		
Leukopenia	Present	11 (11.0%)	30 (17.8%)	36 (25.2%)	7.95	0.019
	Absent	89 (89.0%)	139 (82.2%)	107 (74.8%)		
Leukocytosis	Present	7 (7.0%)	17 (10.1%)	32 (22.4%)	14.89	0.001
	Absent	93 (93.0%)	152 (89.9%)	111 (77.6%)		

## Discussion

This study evaluated hematological alterations in patients with chronic kidney disease (CKD) across stages 3-5 and demonstrated a progressive decline in hemoglobin, hematocrit, red blood cell indices, and platelet counts, accompanied by a rise in red cell distribution width and leukocyte derangements with advancing disease severity. The mean age of the participants in this study was 44.26 ± 14.05 years, with 56.1% between 46 and 70 years, and 64.6% being male. This demographic distribution is younger than many international cohorts. Abdelnabi et al. (2021) [18] reported mean ages of 51.5 ± 14.6 years among Egyptian patients, while Erken et al. (2020) [19] reported a mean age of 63.2 ± 14.9 years. The relatively younger profile in this cohort likely reflects the earlier onset of CKD in the Asian populations due to high burdens of diabetes and hypertension at younger ages. In the present study, diabetes mellitus (40%) and hypertension (31.3%) were the leading etiologies, followed by renal stone disease (15.8%) and glomerular disease (12.9%). These findings are concordant with Afshar et al. (2010), who observed diabetes in 49.1% and hypertension in 28.3% of patients with CKD [20]. Kaze et al. (2020) [12] similarly noted hypertension in 81.9% and diabetes in 35.2%, while Ahmed et al. (2021) [16] observed diabetes in 51.9% and hypertension in 29.5% of patients. This consistency across diverse populations underlines the shared burden of metabolic and hypertensive disorders in driving CKD progression.

In our cohort, the mean Hb level was 10.92 ± 2.05 g/dL, with a progressive decline across stages: 12.65 ± 1.05 g/dL in stage 3, 10.69 ± 1.95 g/dL in stage 4, and 9.98 ± 1.97 g/dL in stage 5 (p < 0.001). Hematocrit showed a similar downward trend (35.61% in stage 3 versus 29.20% in stage 5, p < 0.001). These trends are consistent with Abdelnabi et al. (2021), who observed significantly lower Hb in patients with CKD (10 g/dL) compared to controls (12.4 g/dL), while Behera (2020) [21] noted a mean Hb of 7.76 g/dL in CKD cases versus 13.2 g/dL in controls [18,21]. Panduranga and Perla (2020) found stage-wise Hb decrease from 9.87 g/dL in stage 3 to 8.37 g/dL in stage 5, mirroring the pattern in the present study [22]. Likewise, Khadayate et al. (2020) documented severe anemia (<7 g/dL) in over 60% of stage 5 patients, highlighting the burden of advanced anemia in dialysis cohorts [23]. The anemia prevalence in the present study (38% in stage 3, 68% in stage 4, and 92.3% in stage 5) parallels the deterioration observed in prior studies, including Kaze et al. (2020), who reported 75% anemia in stage 3 and 93.9% in stage 5 [12]. However, the overall mean Hb in our cohort was higher than that reported by Khadayate et al. (7.49 g/dL) [23] and Behera (7.76 g/dL) [21]. This difference may be explained by the exclusion of patients with severe anemia in some studies, local differences in nutritional and iron supplementation, and variable access to the erythropoietin therapy.

In this study, RBC count declined from 4.04 ± 0.74 × 10¹²/L in stage 3 to 3.38 ± 0.50 × 10¹²/L in stage 5 (p < 0.001), with an overall mean of 3.64 ± 0.67 × 10¹²/L. This pattern is seen in Swarnalatha and Vinotha (2020) [12], who reported a stage-wise decrease (3.26 in stage 3 versus 1.38 in stage 5, p < 0.001), and Rahman et al. (2022) [24], who found a reduction from 3.54 in early CKD to 2.35 × 10¹²/L in advanced CKD. These consistent findings across diverse regions reinforce the central pathophysiological role of erythropoietin deficiency and reduced marrow responsiveness in CKD-related anemia. The present study demonstrated significant reductions in mean corpuscular volume (85.14 to 80.13 fL, p = 0.001) and mean corpuscular hemoglobin (30.17 to 27.41 pg, p < 0.001), alongside an increase in RDW (13.58 to 15.79%, p < 0.001). These findings reflect the development of anisocytosis with advancing disease. Behera (2020) [21] reported elevated RDW (16.3 versus 14.5 in controls), while Erken et al. (2020) [19] confirmed significant increases in RDW with advanced CKD, correlating negatively with hemoglobin. Similarly, Khadayate et al. (2020) [23] and Panduranga and Perla (2020) [22] observed RDW elevation in advanced CKD, although statistical significance varied across studies. Together, these findings highlight RDW as a simple yet robust marker of anemia severity, inflammation, and comorbidity in CKD.

In our study, platelet counts declined significantly from 272.15 × 10⁹/L in stage 3 to 197.82 × 10⁹/L in stage 5 (p < 0.001). Thrombocytopenia prevalence increased from 14% to 34.3% across stages, whereas thrombocytosis remained infrequent (6.5%-9.1%) and not significantly associated with stage. These findings align with Behera (2020), who reported lower platelet counts in cases (2.27 lakh/µL) compared to controls (2.45 lakh/µL) [21], and Khadayate et al. (2020) [23], where 43.7% had platelets below 1.5 lakh/µL. Swarnalatha and Vinotha (2020) documented significant platelet count reduction from stage 3 to 5, paralleling the current results [11]. The mechanism of thrombocytopenia in CKD involves reduced thrombopoietin production, uremic toxins impairing megakaryocyte maturation, and increased platelet destruction.

In this research, white blood cell count increased significantly with CKD progression (7.18 ± 2.54 × 10⁹/L in stage 3 versus 8.23 ± 2.64 × 10⁹/L in stage 5, p = 0.005). Neutrophils rose from 4.66 to 5.42 (p < 0.001), while lymphocytes declined from 3.03 to 2.49 (p < 0.001). Consequently, leukocytosis increased from 7% to 22.4%, and leukopenia from 11% to 25.2%, both showing significant stage-wise associations. These findings resonate with Behera (2020) [21], who observed higher leukocyte counts in cases (20.46 × 10⁹/L) versus controls (6.91 × 10⁹/L), and Khadayate et al. (2020) [23], who reported elevated total leukocyte counts in 27.1% and reduced counts in 15.5%. Swarnalatha and Vinotha (2020), however, demonstrated a declining trend in WBC with progression, underscoring heterogeneity across populations [11]. Kaze et al. (2020) found leukopenia in 15.2% and hyperleukocytosis in 6.7%, although without statistical significance [12]. The rise in neutrophils and fall in lymphocytes in the present study mirror the pro-inflammatory milieu of CKD. Ahmed et al. (2021) reported an elevated neutrophil to lymphocyte ratio (3.3) in advanced CKD, reinforcing the prognostic significance of leukocyte shifts in predicting outcomes such as cardiovascular events and mortality [16].

This study is strengthened by its large sample size and comprehensive evaluation of multiple hematological parameters across CKD stages using standardized laboratory methods. It provides robust local data from a tertiary care center, increasing generalizability to similar populations. However, being cross-sectional, it cannot establish temporal or causal relationships. Potential confounders such as nutritional deficiencies, iron status, and erythropoietin therapy were not fully assessed, which may influence hematological outcomes. Future studies should incorporate longitudinal follow-up with mechanistic evaluation, including iron metabolism, inflammatory biomarkers, and treatment effects, to better delineate the progression and clinical implications of hematological abnormalities in CKD.

## Conclusions

This study demonstrates that hematological abnormalities are highly prevalent in patients with chronic kidney disease and worsen significantly with disease progression. Declines in hemoglobin, hematocrit, red blood cell count, and platelets, alongside rising red cell distribution width and leukocyte disturbances, reflect the complex interplay of anemia, inflammation, and bone marrow dysfunction in advanced CKD. Anemia emerged as the most frequent abnormality, followed by thrombocytopenia and leukocyte disorders, with strong statistical associations with later CKD stages. These findings highlight the clinical need for routine hematological monitoring in patients with CKD to facilitate timely intervention, reduce morbidity, and improve overall outcomes.
